# Trends in Prices of Drugs Used to Treat Metastatic Non–Small Cell Lung Cancer in the US From 2015 to 2020

**DOI:** 10.1001/jamanetworkopen.2021.44923

**Published:** 2022-01-25

**Authors:** Aakash Desai, Caleb Scheckel, Chelsee J. Jensen, Jacob Orme, Colt Williams, Nilay Shah, Konstantinos Leventakos, Alex A. Adjei

**Affiliations:** 1 Division of Medical Oncology, Mayo Clinic, Rochester, Minnesota; 2 Department of Finance, Mayo Clinic, Rochester, Minnesota

## Abstract

**Importance:**

Oncology drug prices are a determinant of health disparities in the US and worldwide. Several new therapeutic agents for non–small cell lung cancer (NSCLC) have become available on the US market over the past decade. Although increased competition typically produces lower prices, competition among brand-name oncology drugs has not resulted in lower prices.

**Objective:**

To assess price changes in class-specific brand-name medications used to treat metastatic NSCLC in the US from 2015 to 2020.

**Design, Setting, and Participants:**

This cross-sectional study, conducted from August 13, 2015, to August 13, 2020, used data from the Micromedex Red Book and Medi-Span Price Rx databases. The study sample was limited to 17 brand-name medications used to treat metastatic NSCLC that were available for purchase before January 1, 2019.

**Main Outcomes and Measures:**

The main outcomes were trends over time in average wholesale prices and wholesale acquisition cost unit prices and the correlation in price among the multiple brand-name medications within each therapeutic class (immune checkpoint inhibitors, epidermal growth factor receptor inhibitors, anaplastic lymphoma kinase inhibitors, ROS1 inhibitors, BRAF inhibitors, and MEK inhibitors), measured using the Pearson correlation coefficient. The compounded annual growth rates of different medication costs were compared with the annual inflation rate and the consumer price index for prescription drugs.

**Results:**

For all drug classes, the Pearson correlation coefficient approached 1.0, indicating an increase in drug list prices despite within-class drug competition. The median Pearson correlation coefficient values were 0.964 (range, 0.951-0.994) for immune checkpoint inhibitors, 0.898 (range, 0.665-0.950) for epidermal growth factor receptor inhibitors, 0.999 (range, 0.982-0.999) for anaplastic lymphoma kinase inhibitors, and 0.999 for BRAF and MEK inhibitors. The median compounded annual growth rates for most drug costs were higher than the annual inflation rate and consumer price index for prescription drugs: 1.81% (range, 1.29%-2.13%) for immune checkpoint inhibitors, 2.56% (range, 2.38%-5.26%) for epidermal growth factor receptor inhibitors, 2.46% (range, 1.75%-4.66%) for anaplastic lymphoma kinase and ROS1 inhibitors, and 3.06% (range, 0%-3.06%) for BRAF and MEK inhibitors.

**Conclusions and Relevance:**

In this cross-sectional study, prices of brand-name medications for treatment of NSCLC increased in the US from 2015 to 2020 without evidence of price competition, raising concern about the affordability of promising oncology drugs. These findings suggest that drug pricing reform is needed.

## Key Points

**Question:**

How did the prices of drugs used for treatment of non–small cell lung cancer change between 2015 and 2020, and was there within-class price competition among brand-name medications?

**Findings:**

In this cross-sectional study of 17 brand-name medications used for treatment of metastatic non–small cell lung cancer, drug prices increased between 2015 and 2020, with increases in prices correlating within each drug class. The increase in prices for these medications was greater than the consumer price index for prescription medications and the inflation rate.

**Meaning:**

The price increases of brand-name medications without evidence of price competition raise concern about the affordability of promising oncology drugs.

## Teaser

This cross-sectional study examines trends in costs of brand-name medications for treatment of non–small cell lung cancer and within-class price differences between these medications in the US from 2015 to 2020.

## Introduction

Several new anticancer agents for treating metastatic non–small cell lung cancer (NSCLC) have been introduced to the US market. In capitalistic systems, increased competition is expected to lower prices; however, this has not been the case with competition among brand-name drugs within the same class in oncology.^1^ Breakthroughs in immunology and genomics have led to the availability of multiple immunotherapy and small-molecule inhibitor drugs, often targeting the same pathway for the treatment of NSCLC. Despite this increased availability, little is known about within-class competition and its effects on the prices of these anticancer agents. Because numerous new drugs have been approved for the treatment of NSCLC in recent years, we sought to specifically study the price competition among drugs used to treat this cancer subtype. We evaluated the pattern of price changes for multiple brand-name medications used for treatment of metastatic NSCLC that were contemporaneous in the US market from 2015 to 2020.

## Methods

**Figure 1.**
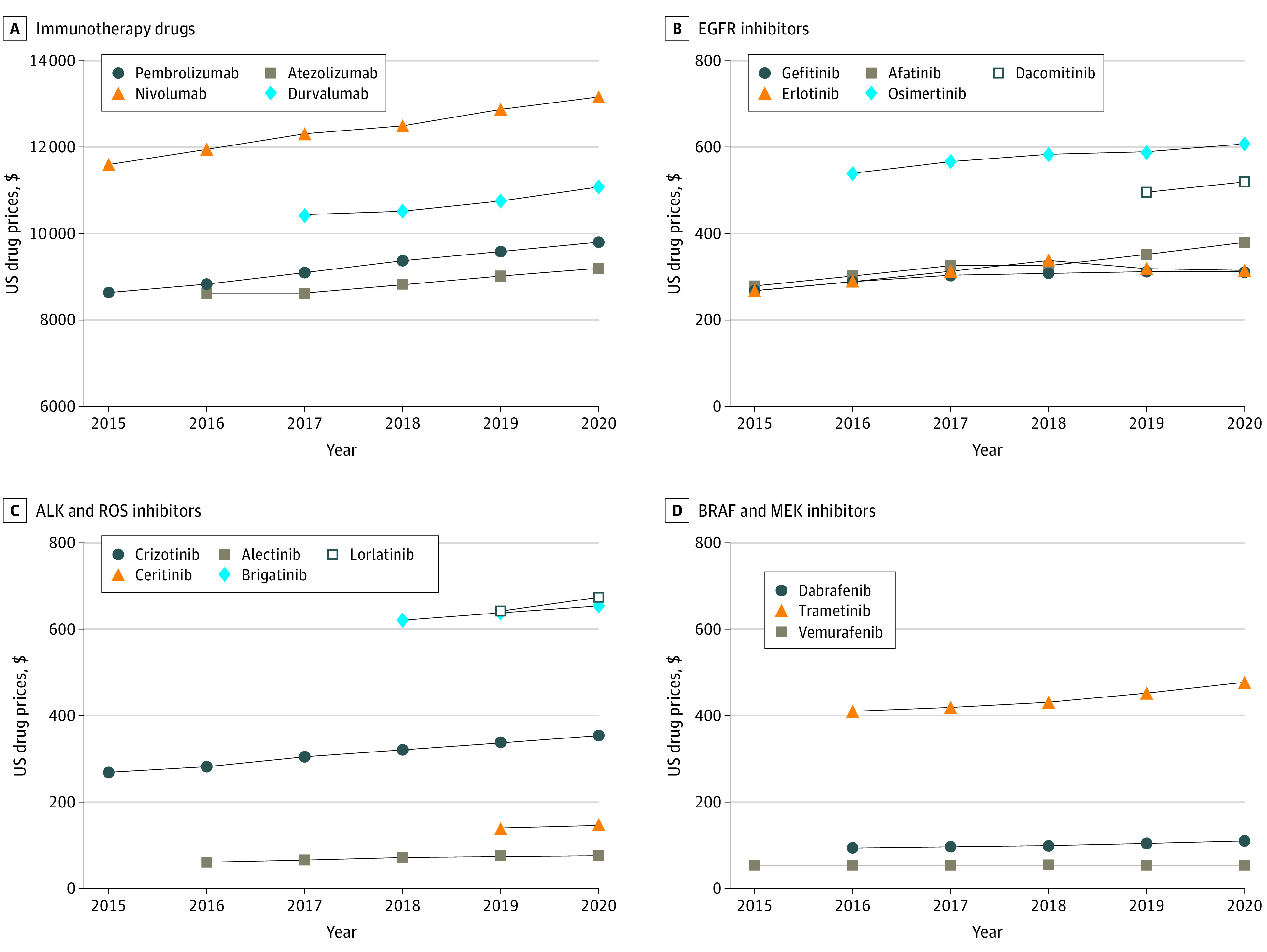
Trends in Prices of Drugs for Metastatic Non–Small Cell Lung Cancer in the US Between 2015 and 2020 ALK indicates anaplastic lymphoma kinase; EGFR, epidermal growth factor receptor.

We conducted a cross-sectional study of average wholesale prices (AWPs) for oral agents and wholesale acquisition cost (WAC) for intravenous agents for treatment of NSCLC in the US from August 13, 2015, to August 13, 2020 (Figure 1). The data were obtained from the Micromedex Red Book and Medi-Span Price Rx databases. The study sample was limited to brand-name medications used for treating metastatic NSCLC that were available for purchase before January 1, 2019, to better characterize the pricing trends of the drugs that have been in the market for more than 1 year. When conflicting drug pricing between Micromedex Redbook and Medi-Span was encountered, the prices provided by Medi-Span were used. Because this study used publicly available data and did not contain any patient- or individual-level data, it was considered to be exempt from institutional review board approval based on the Common Rule. This study followed the Strengthening the Reporting of Observational Studies in Epidemiology (STROBE) reporting guideline.

Average wholesale price describes the average price paid by retail pharmacies for a drug from the wholesaler and is used by insurers to determine the reimbursement of prescription drugs. Wholesale acquisition cost is the manufacturer’s list price when the drug is sold to the wholesaler and subsequently distributed to health care institutions. Average wholesale price and WAC pricing presented in the Table were calculated based on the commonly approved doses and do not include contracts, rebates, or other discounts.

**Table.** Cost Trends for Medications for Metastatic Non–Small Cell Lung Cancer in the US From 2015 to 2020

Drugs	Cost, US $^a^						CAGR, %
	2015	2016	2017	2018	2019	2020	
Immunotherapy							
Pembrolizumab	8632.00	8827.24	9094.12	9369.04	9580.40	9797.00	2.13
Nivolumab	11 596.73	11 947.24	12 308.35	12 492.98	12 870.58	13 160.90	2.13
Atezolizumab	NA	8620.00	8620.00	8814.92	9013.75	9194.03	1.30
Durvalumab	NA	NA	10 436.43	10 514.73	10 752.48	11 077.47	1.50
EGFR inhibitors							
Gefitinib	268.00	289.40	304.00	308.40	312.00	312.00	2.57
Erlotinib	268.30	289.80	313.00	338.10	319.44	315.08	2.71
Afatinib	279.66	302.03	326.20	326.20	352.30	380.50	5.27
Osimertinib	NA	540.60	567.60	584.70	590.50	608.30	2.39
Dacomitinib	NA	NA	NA	NA	496.00	520.80	2.47
ALK and ROS inhibitors							
Crizotinib	269.30	282.80	305.80	321.10	337.20	354.00	4.66
Ceritinib	NA	NA	NA	NA	140.70	146.30	1.97
Alectinib	NA	61.60	66.50	72.50	74.70	76.90	4.54
Brigatinib	NA	NA	NA	621.30	638.60	654.60	1.76
Lorlatinib	NA	NA	NA	NA	642.23	674.34	2.47
BRAF and MEK inhibitors							
Dabrafenib	NA	93.70	96.51	99.31	104.27	110.00	3.26
Trametinib	NA	410.90	419.10	431.30	452.80	477.80	3.06
Vemurafenib	54.25	54.25	54.25	54.25	54.25	54.25	0

Abbreviations: ALK, anaplastic lymphoma kinase; CAGR, compounded annual growth rate; EGFR, epidermal growth factor receptor; NA, not applicable.

^a^ Wholesale acquisition cost and average wholesale price history.

Multiple contemporaneous brand-name medications on the market were assessed in the following therapeutic classes: immune checkpoint inhibitors (ICIs; immunotherapy), epidermal growth factor receptor (EGFR) inhibitors, anaplastic lymphoma kinase (ALK) inhibitors, ROS1 inhibitors, BRAF inhibitors, and MEK inhibitors.

### Statistical Analysis

The primary outcome was the trend over time in AWP and WAC unit prices and the strength of the linear association between prices of drugs among the multiple brand-name medications within each therapeutic class. Price correlations were measured using the Pearson correlation coefficient. In addition, the compound annual growth rates (CAGRs) for brand-name medication costs within each therapeutic class were calculated. Compound annual growth rate denotes the mean annual increase in drug prices. All analyses were performed using the packages *duly* and *stat* in R, version 4.0.3 (R Project for Statistical Computing) and using Excel spreadsheet software, version 14.7.6 (Microsoft).

## Results

**Figure 2.**
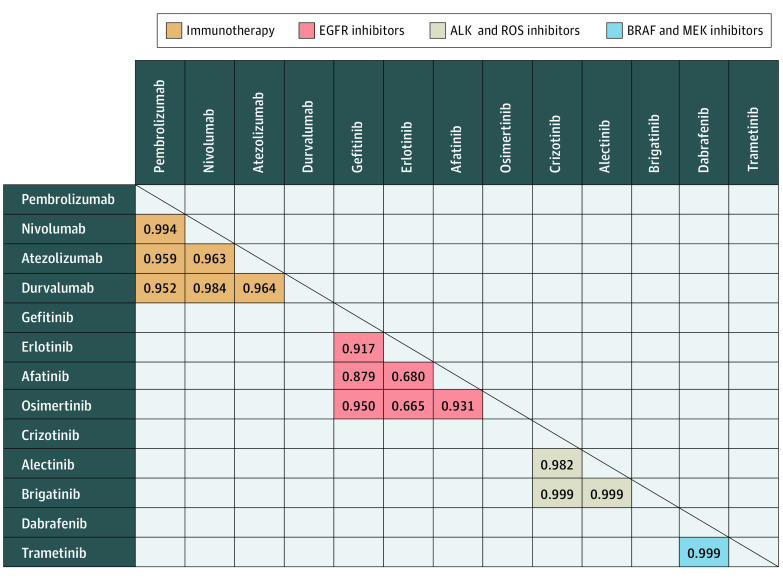
Pearson Correlation Coefficients Among Brand-Name Medications Within the Same Drug Class for Metastatic Non–Small Cell Lung Cancer in the US From 2015 to 2020 ALK indicates anaplastic lymphoma kinase; EGFR, epidermal growth factor receptor.

This study included 17 brand-name drugs: 4 ICIs (pembrolizumab, nivolumab, atezolizumab, and durvalumab), 5 EGFR inhibitors (gefitinib, afatinib, erlotinib, osimertinib, and dacomitinib), 5 ALK inhibitors (crizotinib, ceritinib, alectinib, brigatinib, and lorlatinib), 2 BRAF inhibitors (dabrafenib, vemurafenib), and 1 MEK inhibitor (trametinib) (Table). The median Pearson correlation coefficient values for drugs within each class were 0.964 (range, 0.951-0.994) for ICIs, 0.898 (range, 0.665-0.950) for EGFR inhibitors, 0.999 (range, 0.982-0.999) for ALK inhibitors, and 0.999 for BRAF and MEK inhibitors. For all classes, median Pearson correlation coefficients approaching 1.0 indicated a strong linear correlation between drug prices of different drugs within the same class (Figure 2). A Pearson correlation coefficient could not be calculated for therapies with 2 or fewer data points (dacomitinib, ceritinib, brigatinib, and lorlatinib) or if prices did not change (vemurafenib).

The median cost CAGRs over this 5-year period were 1.81% (range, 1.29%-2.13%) for ICIs, 2.56% (range, 2.38%-5.26%) for EGFR inhibitors, 2.46% (range, 1.75%-4.66%) for ALK and ROS inhibitors, and 3.06% (range, 0%-3.06%) for BRAF and MEK inhibitors. Except for ICIs, the median cost CAGR outpaced the annual growth rate of the consumer price index for prescription drugs at 2.10% and, for all classes, the average yearly inflation rate of 1.75% during the same period. Of note, among all therapeutic classes studied, there was only 1 price decrease. This was observed for erlotinib between 2019 and 2020, and it corresponded with the introduction of a generic competitor to the market.

## Discussion

This cross-sectional study found a positive correlation between high list prices among different drugs within the same class on the market across 5 therapeutic classes used for metastatic NSCLC from 2015 to 2020 in the US. These results suggest that there was little price competition among the manufacturers of these products. This is consistent with previous findings that little difference exists in the median wholesale price of novel drugs and next-in-class drugs.^2^ Although one might expect oncology drug prices to decrease over time after market entry, the list price of most anticancer agents increases paradoxically. An earlier study of 24 patented injectable anticancer agents in the US demonstrated that prices increased by 25% over a period of 8 years after launch; these increases in cost were not offset by supplemental US Food and Drug Administration approvals, new competitors, or new off-label indications.^3^ Thus, price increases over time were not substantially reduced by market competition and increased at similar rates among drugs within the same class.

Among the agents studied, we identified only 1 instance of a decrease in price, which coincided with the introduction of a generic formulation. However, in general, the introduction of generics does not substantially change the cost of cancer therapy.^4^ For example, when generic imatinib was introduced, its monthly sales price was only 8% less than the price of the brand-name imatinib. Despite this small reduction in price, generics offer the promise of lowering prices and making drugs more affordable for patients. Reduction in drug costs over time with the use of generics can improve cost-effectiveness, change reimbursement decisions, and increase the number of treatment options available to patients.^5^ Although generics offer the promise of lowering prices, there are numerous instances when generic competition is delayed or when manufacturers extend patents or develop more convenient dosage forms to keep market share.^6^

With the exception of the immunotherapy class (median CAGR, 1.81%), the median cost CAGR outpaced the annual growth rate of the consumer price index for prescription drugs at 2.10% and, for all classes, the average yearly inflation rate of 1.75% during the same period. This may have been attributable to an increasing number of immunotherapy drugs in the pipeline and an expanding number of indications, which may allow drug manufacturers to keep the prices lower compared with the other targeted agents. Furthermore, given the global approvals of immunotherapy agents, the vast global market share may play a role in the lower CAGRs for this class of drugs.

Although AWP and WAC do not always reflect the true net prices paid by insurers or patients, increasing drug prices correlate with higher out-of-pocket expenses for patients.^7^ Financial toxicity may be associated with increased symptom burden, worse quality of life, and increased cancer mortality.^8^

### Limitations

This study has limitations. Public WAC and AWP pricing does not include any discount negotiations or rebates that occur between wholesalers, institutions, insurers, or pharmacy benefit managers. However, rebates, list prices, and net prices have been increasing for brand-name medications, and rebate growth has been shown to be positively correlated with list price growth, thereby impacting costs for patients.^9^

## Conclusions

This cross-sectional study found that, between 2015 and 2020 in the US, the costs of within-class drugs used to treat metastatic NSCLC correlated closely, with minimal price competition among manufacturers. This may not be the expected outcome in a liberal economy, in which competition should lead to lower prices for consumers. The median change in drug list prices for the medications studied outpaced that of other prescription drugs and the average inflation rate. The lock-step price increases of brand-name medications without evidence of price competition raise concern about the affordability of promising oncology drugs. Academic, industry, and government partnerships should be developed to address the high costs of prescription oncology drugs, which may soon be unaffordable for most patients if the trends discovered in the present study continue.
